# Development of [^111^In]In-CHX-A″-DTPA-αCD68 for ImmunoSPECT to Image Murine Macrophages

**DOI:** 10.1021/acs.molpharmaceut.5c01902

**Published:** 2026-07-02

**Authors:** Anna E. Strong, Erika Belitzky, Ayla Vaughn Embs, Samantha Katz, Alessandra Cavaliere, Dijana Djureinovic, Borna Roohani, Michael Liu, Carla Rothlin, Jianmei W. Leavenworth, Lucia Jilaveanu, Harriet M. Kluger, Bernadette Marquez-Nostra

**Affiliations:** † Department of Radiology, University of Alabama at Birmingham, Birmingham, Alabama 35233, United States; ‡ Yale PET Center, Department of Radiology and Biomedical Imaging, 5755 Yale University, New Haven, Connecticut 06520, United States; § Department of Medical Oncology, 25045 Yale University, New Haven, Connecticut 06520, United States; ∥ Department of Laboratory Medicine and Pathology, University of Minnesota, Minneapolis, Minnesota 55455, United States; ⊥ Department of Neurosurgery, University of Alabama at Birmingham, Birmingham, Alabama 35233, United States; # The O’Neal Comprehensive Cancer Center, University of Alabama at Birmingham, Birmingham, Alabama 35233, United States

**Keywords:** CD68, immunoSPECT imaging, innate, tumor-associated macrophages, CSF1R, renal cell carcinoma

## Abstract

**Purpose:** High tumor-associated macrophage (TAM) abundance is associated with poorer prognoses across many cancers, including renal cell carcinoma (RCC). CD68 is an established clinical biomarker for macrophages in patient tissues and is also expressed in mouse macrophages, making it an attractive target for bridging preclinical and clinical imaging of macrophages. We developed an anti-mouse CD68 (mCD68) immunoSPECT imaging agent to noninvasively quantify TAM burden in vivo and evaluate response to macrophage-modulating treatment in a syngeneic RCC tumor model. **Methods:** [^111^In]­In-CHX-A″-DTPA-αCD68 was synthesized by optimizing chelator conjugation and radiolabeling conditions, then characterized for binding affinity, stability, and specificity by in vitro assays. In vivo specificity was assessed in the Renca syngeneic RCC model by comparing biodistribution of [^111^In]­In-CHX-A″-DTPA-αCD68 with that of the radiolabeled IgG isotype control. ImmunoSPECT signal was correlated with ex vivo CD68 expression measured by flow cytometry of dissociated tumors and spleens. To evaluate response to macrophage-modulating treatment, a 2 × 2 factorial study was conducted in which Renca-allografted mice received either anti-CSF1R or IgG control, followed by immunoSPECT imaging with [^111^In]­In-CHX-A″-DTPA-αCD68 or radiolabeled isotype control; organ-to-heart SUVR was correlated with ex vivo CD68 expression by flow cytometry. **Results:** [^111^In]­In-CHX-A″-DTPA-αCD68 bound specifically to CD68 in bone marrow-derived macrophages, with high binding affinity (K_D_ = 7.99 ± 2.26 nM) to the CD68 protein. In Renca-allografted mice, immunoSPECT signal correlated strongly with ex vivo CD68 expression measured by flow cytometry of dissociated tumor and spleen cells (*r* = 0.854). Biodistribution studies confirmed specific uptake in vivo, with 2-fold, 8-fold, and 8-fold greater uptake in tumor, spleen, and bone marrow, respectively, compared with the radiolabeled isotype control. Treatment with the macrophage-depleting anti-CSF1R resulted in ∼40% lower tumor-to-heart SUVR with [^111^In]­In-CHX-A″-DTPA-αCD68 compared with the isotype control, despite no change in tumor volumes. ImmunoSPECT signal remained strongly correlated with ex vivo CD68 expression measured by flow cytometry (*r* = 0.952). **Conclusions:** This study demonstrates the first example of immunoSPECT imaging of CD68 in vivo. ImmunoSPECT imaging of CD68 provides a direct, noninvasive measure of total TAM burden in RCC and represents a translatable approach for monitoring response to macrophage-modulating treatments in preclinical studies.

## Graphical abstract

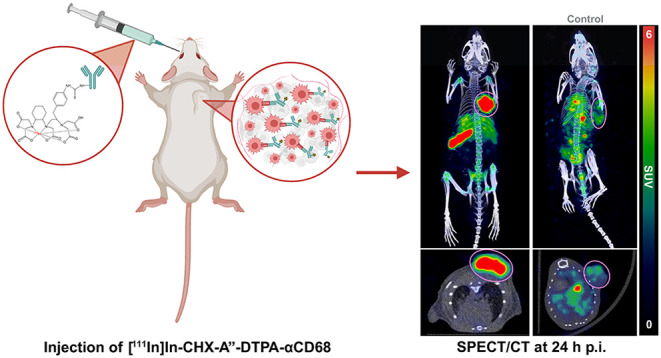

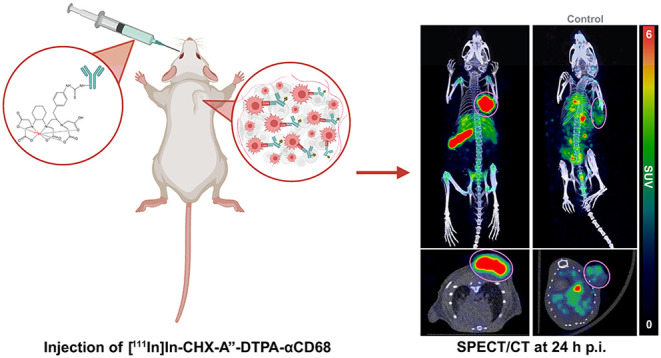

## Introduction

Macrophages are innate immune cells involved in the pathogenesis of numerous diseases, including cancer, cardiovascular disease, and neurodegenerative disease.^1^ In cancer, tumor-associated macrophages (TAMs) can reduce the effectiveness of chemotherapy, radiotherapy, and immunotherapy by promoting tumor cell motility, angiogenesis, proliferation, and immune evasion. TAMs comprise the bulk of some tumor masses, sometimes making up over 50% in renal cell carcinoma (RCC) tumors.^2^ High TAM abundance is also associated with poorer prognoses across many cancers, including RCC.^3^ Several clinical trials are investigating TAM-targeted therapies, including combination strategies targeting CD40 and CSF1R.^4–8^ While CD40 activation promotes macrophages to secrete pro-inflammatory cytokines to destroy tumor cells, anti-CSF1R depletes macrophages but can also change their function to reduce tumor growth.^7^ TAMs can exhibit plasticity in response to tumor-derived and tissue-derived signals; therefore, they cannot be strictly classified as proinflammatory and anti-inflammatory.^8^ These functional changes underscore the need for noninvasive imaging to monitor the total macrophage density and functional state within the tumor microenvironment (TME).

CD68 is an established clinical biomarker of macrophages, overexpressed in macrophages relative to other cell types.^9^ Its expression across all macrophage populations, regardless of functional state, makes it suitable for quantifying the total TAM burden in vivo. CD68 is a heavily glycosylated protein and a member of the lysosomal/endosomal-associated membrane glycoprotein family.^10,11^ CD68 is predominantly located in the lysosomes and endosomes of the cell but can rapidly shuttle to the cell surface, enabling access to antibody-based targeting.

Given that mouse models with intact immune systems are essential for the preclinical evaluation of TAM-modulating therapies, and the moderate homology between mouse and human CD68,^11^ a new imaging probe targeting mouse CD68 is needed to support translational relevance and bridge the gap between preclinical studies and clinical outcomes. Therefore, we developed an anti-mouse CD68 (mCD68) immunoSPECT probe to noninvasively quantify total macrophage density, using a syngeneic tumor model of renal cell carcinoma as a proof of concept investigation.

To understand the role of macrophages and the effects of macrophage-targeted treatments in cancer, immunohistochemical (IHC) staining of CD68 is the gold standard for tissue-based analysis of macrophages.^11^ However, to obtain a longitudinal and three-dimensional picture of total macrophage burden in vivo, immunoSPECT imaging of macrophages with radiolabeled anti-CD68 is a noninvasive approach for tracking these immune cells in vivo. Indium-111 (t_1/2_ = 2.8 days) is well matched to the biological half-life of intact antibodies. SPECT offers the additional advantage of multiplex imaging, enabling simultaneous detection of multiple radioisotopes with distinct gamma signatures.^12^ Herein, we describe the development, characterization, and immunoSPECT application of an ^111^In-labeled anti-mCD68. This approach will be particularly useful for the translation of anti-CD68 immunoSPECT imaging of macrophages to clinical studies using an anti-human CD68 variant, to complement or overcome the limitations of invasive biopsy-based pathological analyses of CD68 in tissues.

## Materials and Methods

### Isolation and Authentication of Bone Marrow-Derived Macrophages

Isolation and differentiation of bone marrow-derived macrophages (BMDMs) were conducted following an established method (Supporting Information Figure S1).^13^ Following differentiation, the analysis of surface CD68 expression on live BMDM cells by flow cytometry was conducted. Cell staining and analysis followed a previously established protocol.^14^

### Validation of CD68 Expression on BMDMs

CD68 expression on BMDMs was validated by immunofluorescence staining with an anti-CD68-Alexa Fluor 546 conjugate, prepared using supplier instructions (Invitrogen, Carlsbad, CA). The rat anti-CD68 antibody used in this experiment, and all following experiments, was clone FA-11 (137002, BioLegend, San Diego, CA). Blocking of CD68 with excess unconjugated anti-CD68 was also performed to evaluate the antibody’s specificity.

BMDMs were fixed in 4% paraformaldehyde (PFA) in 6-well plates containing glass coverslips. Cells were washed with PBS, blocked with 1% bovine serum albumin (BSA) in PBS containing 0.05% tween-20 (PBST) for 30 min, then incubated with 2 μg/mL of anti-CD68-Alexa Fluor 546 for 1 h at ambient temperature in a humidified chamber. For blocking studies, a final concentration of 2 mg/mL (1000-fold excess) of unconjugated anti-CD68 was added prior to the addition of the conjugate. Cells were washed thrice with PBS, and counter-stained with 4′,6-diamidino-2-phenylindole (DAPI, 1:250, ThermoFisher, Waltham, MA) for 15 min at ambient temperature. Coverslip-mounted slides were imaged at excitation/emission wavelengths of 554/570 nm using EVOS (ThermoFisher).

### Synthesis of ^111^In-labeled Antibodies

^111^In-labeled anti-mCD68 was synthesized as the immunoSPECT imaging probe for mouse macrophages. ^111^In-labeled IgG isotype control was also prepared as a negative control. Rat anti-mCD68 and IgG2a isotype control (BP0089, BioXcell, Lebanon, NH) antibodies were conjugated and radiolabeled using trace-metal-grade reagents and supplies. The initial concentrations for each antibody were approximately 5 mg/mL for the anti-CD68 and 8 mg/mL for the IgG. Each antibody was conjugated to a 20-fold molar excess of [(R)-2-amino-3-(4-isothiocyanatophenyl)­propyl]-trans-(S,S)-cyclohexane-1,2-diamine-pentaacetic acid (CHX-A″-DTPA, B-355, Macrocyclics, Plano, TX) in 50 mM Na_2_CO_3_ buffer (pH 9) for 1 h at 37 °C in a thermomixer, shaking at 800 rpm to obtain CHX-A″-DTPA-anti-CD68 or CHX-A″-DTPA-IgG2a. Unconjugated chelate was removed by double buffer exchange using a 40K MWCO Zeba spin desalting column with 50 mM NH_4_OAc (pH 5.5). The conjugates were radiolabeled with indium-111 chloride (^111^InCl_3_, BWXT Medical, Vancouver, Canada) under the following conditions: a final concentration of 1 mg/mL, pH 5.5, specific activity of 370 MBq/mg, and incubation at 37 °C for 1 h with shaking to obtain [^111^In]­In-CHX-A″-DTPA-anti-CD68 or [^111^In]­In-CHX-A″-DTPA-IgG2a.

Radiochemical yield was determined by radio-instant thin-layer chromatography (radio-iTLC) using 50 mM DTPA (pH 7) as the mobile phase. Antibody purity was determined by radio-size-exclusion high-performance liquid chromatography (radio-SEC-HPLC) equipped with a TSKgel G2000SWxl column (0008540, Tosoh Biosciences, Tokyo, Japan), using an Agilent 1260 (Santa Clara, CA). An isocratic gradient was applied by using a mobile phase consisting of 0.2 M NaH_2_PO_4_ and 0.2 M NaCl buffer (pH 7) at a flow rate of 1 mL/min. ^111^In-labeled antibodies were buffer-exchanged into PBS, as described above, prior to their use in subsequent in vitro and in vivo studies.

### Culturing of Cancer Cells

Renca, a murine RCC cell line, was cultured according to protocols established by ATCC (Manassas, VA).^15^ Renca cells were also authenticated by STR profiling (ATCC) and tested for mycoplasma twice over the course of the studies. Passage numbers of 20–25 were used.

### In Vitro Studies

The biophysical integrity of [^111^In]­In-CHX-A″-DTPA-αCD68 over time was tested in mouse serum (M5905, Sigma, St. Louis, MO) and in PBS at radioactivity doses greater than those used for animal studies to evaluate potential radiolysis. Aliquots of 18.5 MBq (500 μCi) [^111^In]­In-CHX-A″-DTPA-αCD68 were diluted in PBS or in mouse serum to a total volume of 500 μL (*n* = 3 per group). The radioconjugate in PBS was incubated at ambient temperature without shaking to determine the expiration time for use in subsequent in vitro and in vivo studies. The radioconjugate in mouse serum was incubated at 37 °C with shaking to assess stability under physiological conditions in vitro. At the end of synthesis (t_0_), 4-, 24-, 48-, and 72-h of incubation, 1.85 MBq (50 μCi) of each aliquot was assayed by radio-SEC-HPLC as described above. The percentage of intact [^111^In]­In-CHX-A″-DTPA-αCD68 monomer was determined by calculating the percentage of monomer relative to the total area under the curve of the peaks in the radiochromatogram. The binding affinity (K_D_) of [^111^In]­In-CHX-A″-DTPA- αCD68 was determined using a radioligand saturation assay with unlabeled anti-CD68, respectively, on immobilized mCD68 recombinant protein (50730-M08B, Sino Biological, Houston, TX) following previous methods.^16^

The specificity of [^111^In]­In-CHX-A″-DTPA-αCD68 for BMDMs over Renca cells was determined by comparing radioligand binding under conditions of nonblocking and CD68-blocking. BMDMs and Renca cells were fixed in 4% PFA in 24-well plates (3.0 × 10^5^ cells per well), and nonspecific sites were blocked with 1% BSA in PBST at ambient temperature for 30 min with shaking. A final concentration of 20 ng/mL of [^111^In]­In-CHX-A″-DTPA-αCD68 was added to the cells (*n* = 3 per group). For CD68-blocking conditions (n = 3 per group), 20 μg/mL of unconjugated anti-mCD68 was added to the cells prior to the addition of the radioconjugate. Plates were incubated for 2 h at ambient temperature with shaking at 200 rpm. Cells were subsequently washed thrice with 1 mL of 1% BSA in PBST to remove unbound antibodies and then lysed with two treatments of 0.5 mL of 1 N sodium hydroxide (NaOH). Lysates were collected in microcentrifuge tubes and assayed in a 2470 Wizard^2^ gamma counter (PerkinElmer, Waltham, MA).

### Tumor Model

All animal studies were performed in accordance with an approved Institutional Animal Care and Use Committee protocol (IACUC-22758). All mice were purchased from Charles River Laboratories (Wilmington, MA) at 7–9 weeks of age. Renca allograft models were generated by inoculating male and female BALB/c mice subcutaneously in the shoulder with 100 μL of 5 × 10^5^ Renca cells in PBS. Mice were selected for studies when their tumor volumes reached at least 50 mm^3^.

### ImmunoSPECT Imaging and Biodistribution Studies

All mice utilized for in vivo studies were injected with 16.1 ± 3.67 MBq of radioconjugate. To evaluate the in vivo specificity of the radioconjugate, male Renca tumor-bearing mice were injected with either [^111^In]­In-CHX-A″-DTPA-αCD68 (*n* = 3) or [^111^In]­In-CHX-A″-DTPA-IgG2a (*n* = 3) via the retro-orbital sinus, followed by SPECT imaging at 24 h postinjection (p.i.). Under 1.5% isoflurane anesthesia, whole-body SPECT images were acquired for 30 min, followed by CT acquisition in a Vector μSPECT/CT scanner (MILabs, Houten, Netherlands). SPECT/CT data were reconstructed using the iterative Similarity-Regulated Ordered-Subset Expectation-Maximization (SROSEM) algorithm, using 0.08 mm voxel size for CT and a 0.8 mm voxel size for SPECT. A calibration factor was applied to SPECT images to translate voxel intensity to activity concentrations in μCi/mL. Activity concentrations were decay-corrected to the date of injection. Tracer uptake in the TME and normal organs was quantified by segmentation on SPECT/CT images using Imalytics Preclinical analysis software (Gremse-IT GmbH, Aachen, Germany). Uptake in the TME and spleen was determined as an organ-to-heart Standardized Uptake Value Ratio (SUVR). The heart was used as the reference organ to account for blood pool and normal CD68 expression in cardiac tissue.^17^ Heterogeneity of uptake in the tumors was quantified, using previous methods, by voxel-based histogram analysis of tumor segmentations and obtaining an SUV heterogeneity index (HI_SUV_).^18,19^ Tumors and spleens were harvested after imaging for flow cytometry studies as described below.

To assess biodistribution ex vivo, a separate cohort of Renca tumor-bearing mice, representing equal numbers of male and female groups, was injected via the retro-orbital sinus with either [^111^In]­In-CHX-A″-DTPA-αCD68 (*n* = 6) or its isotype control [^111^In]­In-CHX-A″-DTPA-IgG2a (*n* = 6). At 24 h p.i., animals were euthanized, and tissues were harvested, weighed, and assayed in a gamma counter. The percentage of injected dose per gram of organ (% ID/g) was calculated.

### Flow Cytometry

Tumors and spleens were processed by enzymatic digestion according to previously established methods.^20^ Tissue dissociation was performed using the gentleMACS Octo Dissociator with Heaters (130–134–029, Miltenyi Biotec, Bergisch Gladbach, Germany) per the manufacturer’s instructions. Single-cell suspensions were filtered through a 70 μm mesh strainer, centrifuged, and subjected to red blood cell lysis (1 mL, 00–4333–57, ThermoFisher) for 2 min, then resuspended in cell freezing media to store single-cell suspensions in −80 °C until staining was performed. After thawing and centrifuging, the single-cell suspensions were resuspended in cell staining buffer (420201, BioLegend). Cells were blocked with rat anti-mouse CD16/CD32 (Mouse BD Fc Block, 553141, BD, Franklin Lakes, NJ) and stained with AF700-CD45 (1:1000, 560693, BD), BV421-CD68 (1:500, 566388, BD), and LIVE/DEAD Fixable Aqua Dead Cell Stain Kit (1:1000, L34957, Invitrogen). TAMs were identified as CD68^+^ CD45^+^ live cells using an Invitrogen Attune NxT Flow Cytometer (ThermoFisher). Tumor single-cell suspensions were gated on forward and side scatter to remove debris, followed by doublet discrimination to isolate single cells. Live cells were selected using a viability dye, and CD45^+^ leukocytes were gated to define tumor-infiltrating immune cells. The gating strategy can be found in Supplementary Figure S2.

### Macrophage Modulating Therapy Study

To evaluate the uptake of the probe with a macrophage-modulating therapy, we conducted an anti-CSF1R treatment study followed by SPECT imaging in the Renca syngeneic tumor model. Following a previously established method with minor modifications, mice were treated with 400 μg of either anti-CSF1R (BE0213, BioXcell) or IgG2a control (BE0089, BioXcell), concurrently with the inoculation of Renca.^21^ Following the initial treatment, mice received therapy every 72 h up to Day 13. On Day 14, mice were injected with either [^111^In]­In-CHX-A″-DTPA-αCD68 (*n* = 4 for anti-CSF1R-treated, *n* = 5 for IgG2a-treated) or [^111^In]­In-CHX-A″-DTPA-IgG2a (*n* = 5 per treatment group). Mice were imaged via SPECT/CT as described above. Tumors were harvested and subjected to flow cytometry analysis as described above.

### Statistical Analysis

Data were analyzed using Prism version 10.2.3 (GraphPad, San Diego, CA) and expressed as mean ± standard deviation, where appropriate. Statistical tests are described individually in the Results section. *P* < 0.05 was considered statistically significant.

## Results

### CD68 is Expressed at High Levels in BMDMs

Through fluorescence imaging, the αCD68-Alexa Fluor 546 showed greater binding to CD68^+^ BMDMs under nonblocking conditions (Figure 1a) compared to CD68-blocking conditions (Figure 1b) at 6 days after differentiation. Analysis of surface CD68 expression on live BMDM cells by flow cytometry revealed that CD68 levels were gradually increased following the differentiation and maturation of BMDMs (Figure 1c–e).

**Figure 1:**
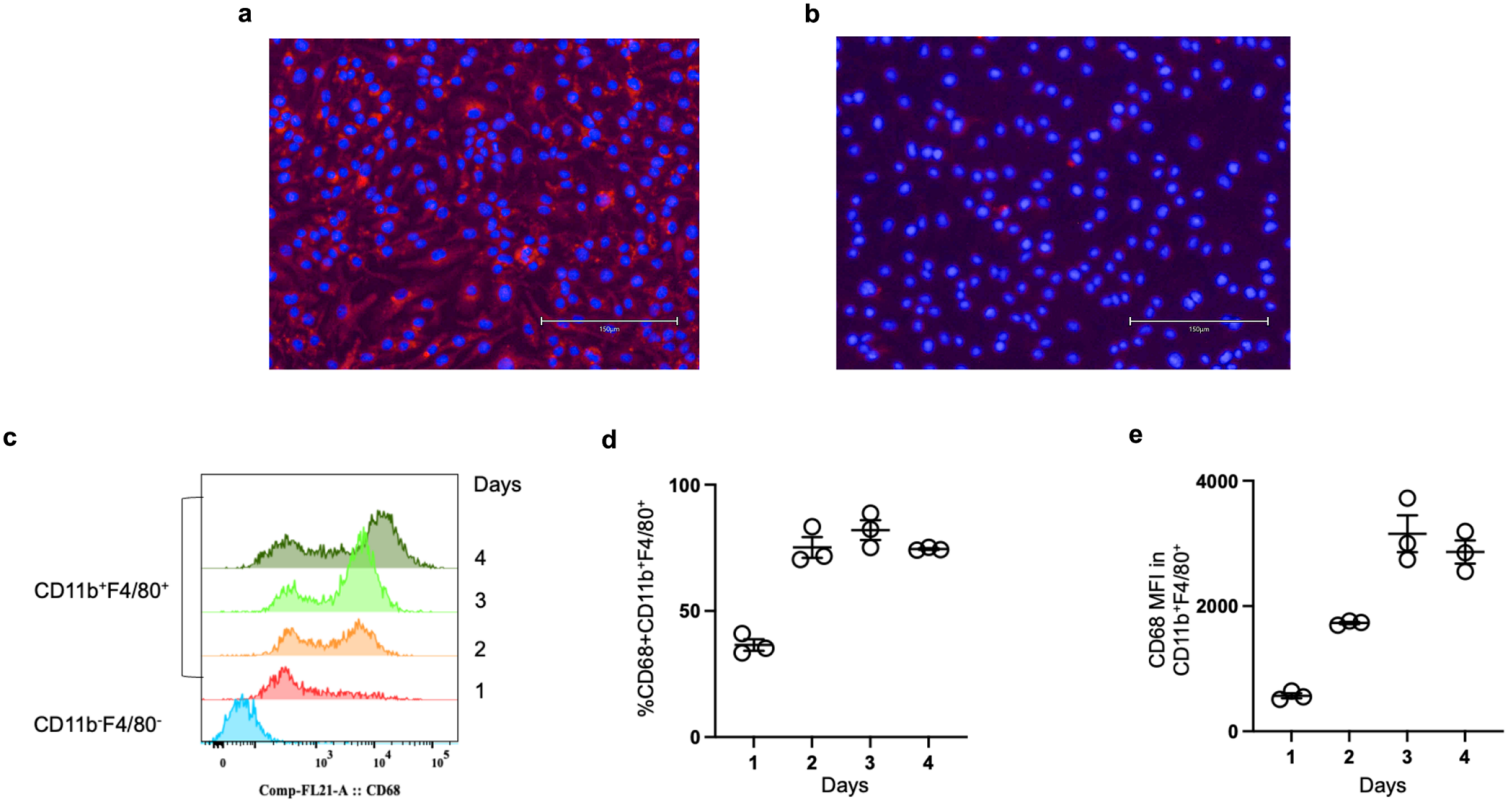
Fluorescence imaging of cell surface CD68 on BMDMs with αCD68-Alexa Fluor 546 conjugate under (a) nonblocking and (b) blocking with 1000-fold unconjugated αCD68. Scale bars represent 150 μm. Red staining represents CD68 expression. Blue staining represents cell nuclei. In addition, flow cytometry studies of macrophage markers under nonpermeabilizing conditions show the representative histogram overlay of CD11b^+^F4/80^+^ BMDMs against mCD68 (c), frequency (d) and expression (e, as indicated as median fluorescence intensity; MFI) of surface CD68 on CD11b^+^F4/80^+^ cells on each day following the differentiation of freshly isolated BMDMs.

### ^111^In-labeled Antibodies were Synthesized with High Specific Activity

[^111^In]­In-CHX-A″-DTPA-αCD68 was synthesized with a specific activity of 370 MBq/mg and a radiochemical yield of ≥95% as determined by radio-iTLC (Supplementary Figure S3a). Radio-SEC-HPLC chromatograms show 98% monomer of the radioconjugate (Supplementary Figure S3b). The same conjugation and radiolabeling conditions were applied to the isotype control antibody, [^111^In]­In-CHX-A″-DTPA-IgG2a. This variant also had a specific activity of 370 MBq/mg and a radiochemical yield of ≥ 95% (Supplementary Figure S3c).

### [^111^In*]*In-CHX-A″-DTPA-αCD68 Shows Stability, High Binding Affinity, and Specificity for CD68 In Vitro

[^111^In]­In-CHX-A″-DTPA-αCD68 was >90% stable in mouse serum at 37 °C and in PBS at ambient temperature up to 72 h. (Figure 2a). No radiolysis was observed at these time points or at radioactivity concentrations double those used for animal studies. These results confirm the physicochemical stability of the radioconjugate. We also determined the binding affinity of [^111^In]­In-CHX-A″-DTPA-αCD68 for CD68 with a K_D_ value of 7.99 ± 2.26 nM (Figure 2b). Nonspecific binding was minimal at all concentrations tested. These results show that [^111^In]­In-CHX-A″-DTPA-αCD68 is suitable for immunoSPECT applications. Binding of [^111^In]­In-CHX-A″-DTPA-αCD68 to CD68^+^ BMDMs was approximately 10-fold higher (64.7% ± 2.19) than under CD68-blocking conditions (6.34% ± 1.05, *p* < 0.0001) and approximately 6-fold higher than binding to Renca cells under nonblocking conditions (10.0% ± 0.53, *p* < 0.0001), confirming specificity for CD68 (Figure 2c). Although a small but statistically significant difference was observed between nonblocking and blocking conditions on Renca cells (p = 0.0171), absolute binding was minimal relative to BMDMs. All comparisons were made by two-way ANOVA.

**Figure 2:**
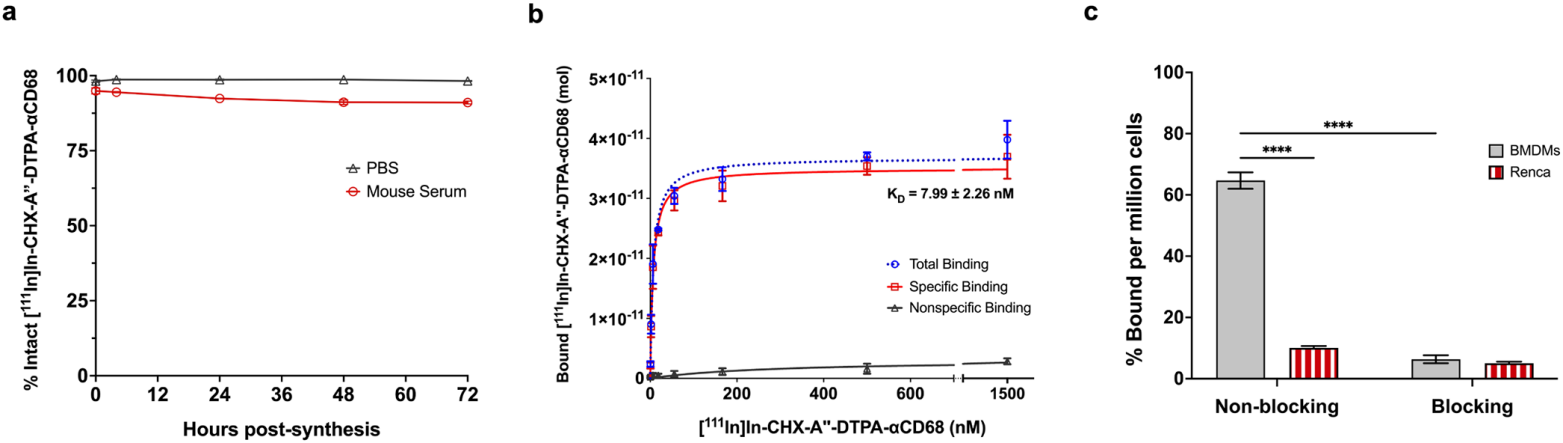
In vitro characterization of [^111^In]­In-CHX-A″-DTPA-αCD68. (a) Stability studies in vitro (*n* = 3 independent assays). Error bars are applied on the graph but are shown to be minimal. (b) Saturation plot showing binding affinity (*n* = 2 independent assays in triplicate wells per assay). (c) Radioligand binding assay on BMDMs compared to Renca cells shows specificity to CD68 and enriched CD68 expression. Significant differences at *p* < 0.0001 are indicated by **** (*n* = 3 per group).

### In Vivo ImmunoSPECT Imaging and Ex Vivo Biodistribution and Flow Cytometry Confirm Specificity

Representative whole-body maximum intensity projections and axial SPECT/CT images are shown in Figure 3a. Shown by a *t*-test, tumor-to-heart SUVR was significantly higher for [^111^In]­In-CHX-A″-DTPA-αCD68 (8.74 ± 1.11) than for the isotype control (0.58 ± 0.13; p = 0.0005; Figure 3b). Voxel-based histogram analysis revealed greater intratumoral heterogeneity of uptake with [^111^In]­In-CHX-A″-DTPA-αCD68 (HI_SUV_ = 2.60 ± 0.53) compared with the isotype control (HI_SUV_ = 2.09 ± 0.08; Figure 3c), consistent with the heterogeneous distribution of TAMs within the tumor.

**Figure 3:**
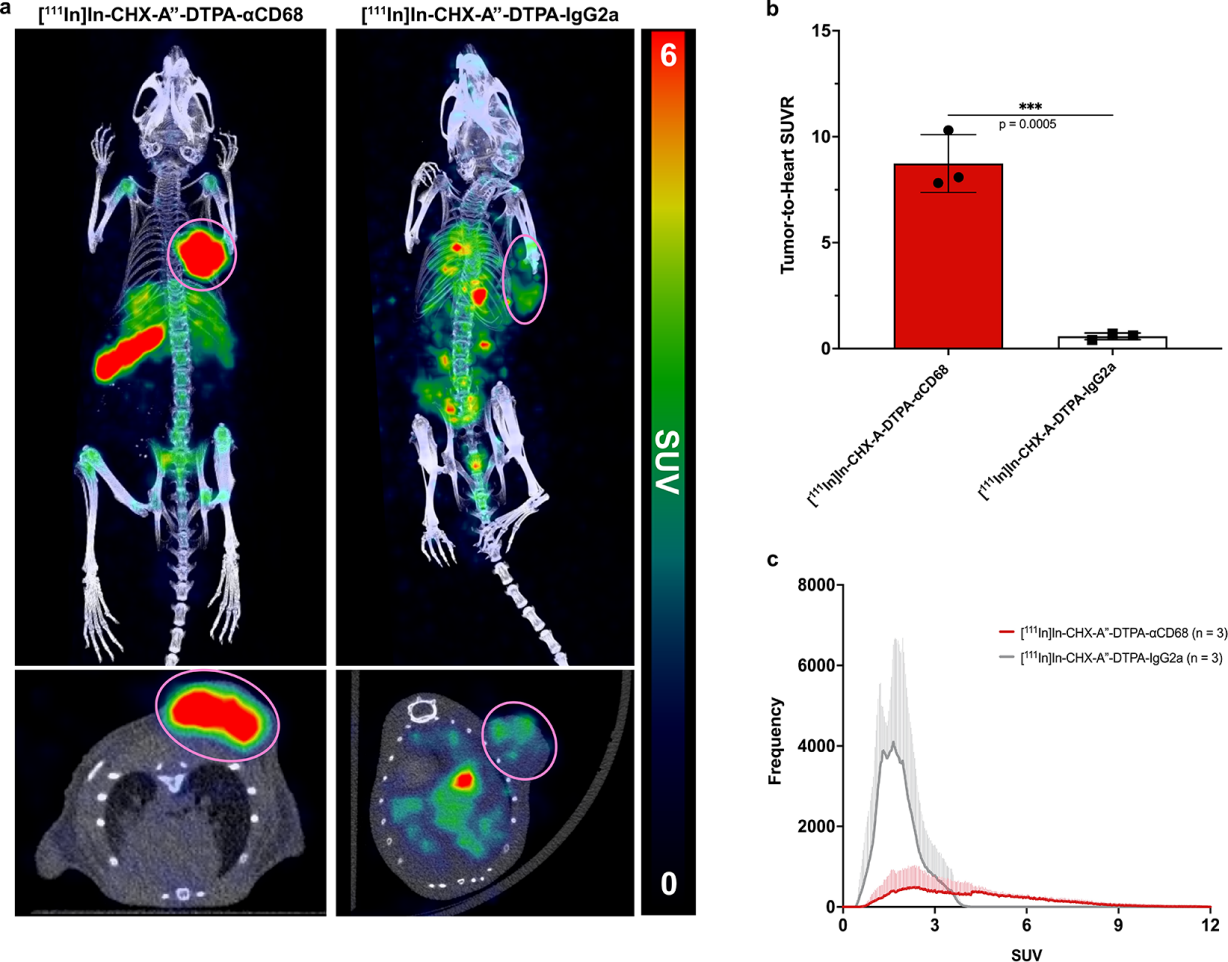
ImmunoSPECT with [^111^In]­In-CHX-A″-DTPA-αCD68 and [^111^In]­In-CHX-A″-DTPA-IgG2a (a) SPECT/CT imaging at 24 h p.i. in the Renca allograft model, showing full-body and axial views. Tumors are circled in pink. (b) Differences in tumor-to-heart SUVR between probes (*p* = 0.0005). (c) Tumor voxel intensity histograms derived from tumor segmentations for each probe.

Spleen-to-heart SUVR was also significantly higher for [^111^In]­In-CHX-A″-DTPA-αCD68 than the isotype control (*p* < 0.0001; Figure 4a). Ex vivo flow cytometry confirmed cell surface CD68 expression, with a two-way ANOVA showing significantly higher CD68 MFI (2750 ± 205) in spleens than in tumors (541 ± 156; *p* < 0.0001; Figure 4b). The absence of significant differences in CD68 expression between probes in either tissue indicates that the injected dose does not deplete CD68^+^ macrophages in vivo, a finding further supported by comparable CD68^+^ TAM levels in tumor-bearing mice that did not receive ^111^In-labeled antibody (Supplementary Figure S4). In mice imaged with [^111^In]­In-CHX-A″-DTPA-αCD68, tumor-to-heart and spleen-to-heart SUVR correlated strongly with CD68 MFI in CD45^+^ live cells (Pearson *r* = 0.854; Figure 4c), indicating that immunoSPECT signal reflects ex vivo CD68 expression.

**Figure 4:**
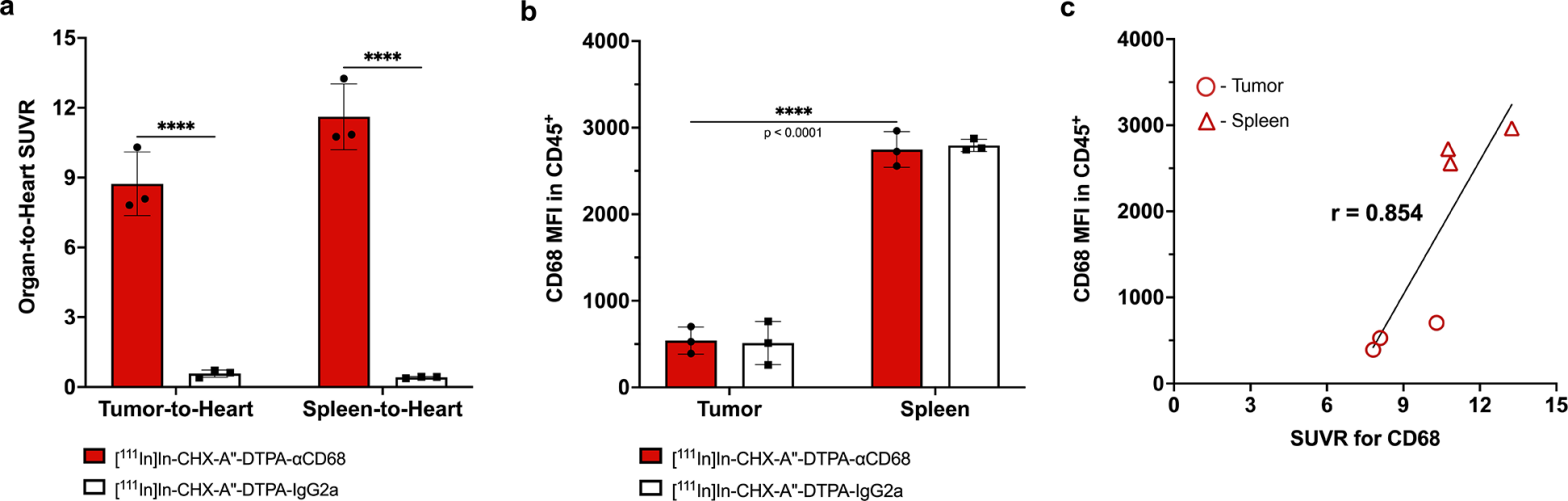
Quantitative relationship between CD68-SPECT and CD68 expression by flow cytometry. (a) Comparison of tumor-to-heart and spleen-to-heart SUVR for [^111^In]­In-CHX-A″-DTPA-αCD68 and [^111^In]­In-CHX-A″-DTPA-IgG2a. (b) Expression of cell surface CD68 by flow cytometry, quantified as CD68 MFI in CD45^+^ live cells. No statistically significant differences were found between probes for the tumors or spleens. (c) Correlative analysis of SUVR and CD68 MFI in CD45^+^ live cells for mice imaged with [^111^In]­In-CHX-A″-DTPA-αCD68 (Pearson *r* = 0.854).

Uptake of [^111^In]­In-CHX-A″-DTPA-αCD68 or [^111^In]­In-CHX-A″-DTPA-IgG2a was compared in normal organs and tumors from biodistribution studies of male and female Renca-allografted mice to determine specificity in vivo. Statistical analysis was conducted via multiple unpaired *t*-tests of the % ID/g of organs between each group. Statistically significant differences in uptake were observed in the lungs, heart, tumor, blood, spleen, muscle, and bone marrow (Figure 5). There were no significant differences in [^111^In]­In-CHX-A″-DTPA-αCD68 uptake based on sex (*p* > 0.05, Supplementary Figure S5). [^111^In]­In-CHX-A″-DTPA-αCD68 had a mean tumor-to-heart ratio (THR) of 5.19 ± 0.92, whereas [^111^In]­In-CHX-A″-DTPA-IgG2a had a significantly lower (*p* = 0.0073) mean THR of 1.61 ± 0.40.

**Figure 5:**
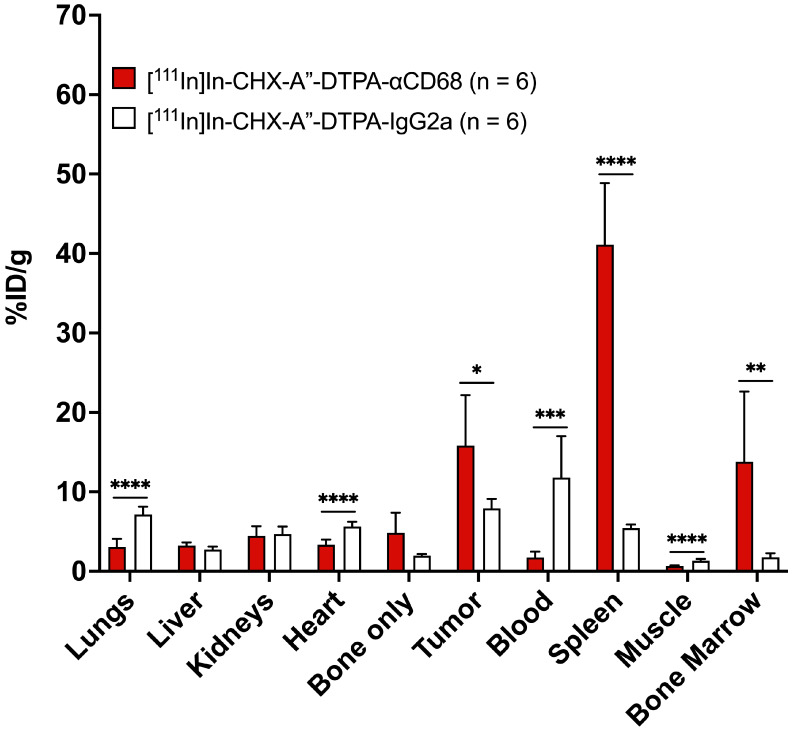
Biodistribution study for [^111^In]­In-CHX-A″-DTPA-αCD68 and [^111^In]­In-CHX-A″-DTPA-IgG2a in Renca allograft model. Significant differences at *p* < 0.05 are indicated by *, *p* < 0.01 indicated by **, *p* < 0.001 indicated by ***, and *p* < 0.0001 indicated by ****.

### ImmunoSPECT Imaging Reveals Macrophage Depletion without Tumor Regression, Validated by Flow Cytometry

To determine whether [^111^In]­In-CHX-A″-DTPA-αCD68 could detect changes in TAM burden following macrophage-modulating treatment, we imaged Renca-allografted mice treated with anti-CSF1R or IgG control. Statistical comparisons in Figure 6a–c were conducted by a two-way ANOVA. Tumor volumes did not differ significantly between treatment groups or imaging probes (Figure 6a). Tumor growth curves can be found in Supplementary Figure S6. In mice imaged with [^111^In]­In-CHX-A″-DTPA-αCD68, tumor-to-heart SUVR was 39.9% lower in anti-CSF1R-treated mice than in IgG controls (p = 0.0380; Figure 6b), with no corresponding difference in mice imaged with the isotype control. Ex vivo flow cytometry confirmed that % CD68^+^ CD45^+^ TAMs were significantly lower in anti-CSF1R-treated mice (3.58 ± 0.33) than in IgG controls (10.5 ± 4.43; p = 0.0340; Figure 6c), with no significant difference between treatment groups for the isotype control. Tumor-to-heart SUVR correlated very strongly with ex vivo surface CD68 expression in mice imaged with [^111^In]­In-CHX-A″-DTPA-αCD68 (Pearson *r* = 0.952; Figure 6d), confirming that immunoSPECT signal quantitatively reflects TAM burden in vivo.

**Figure 6:**
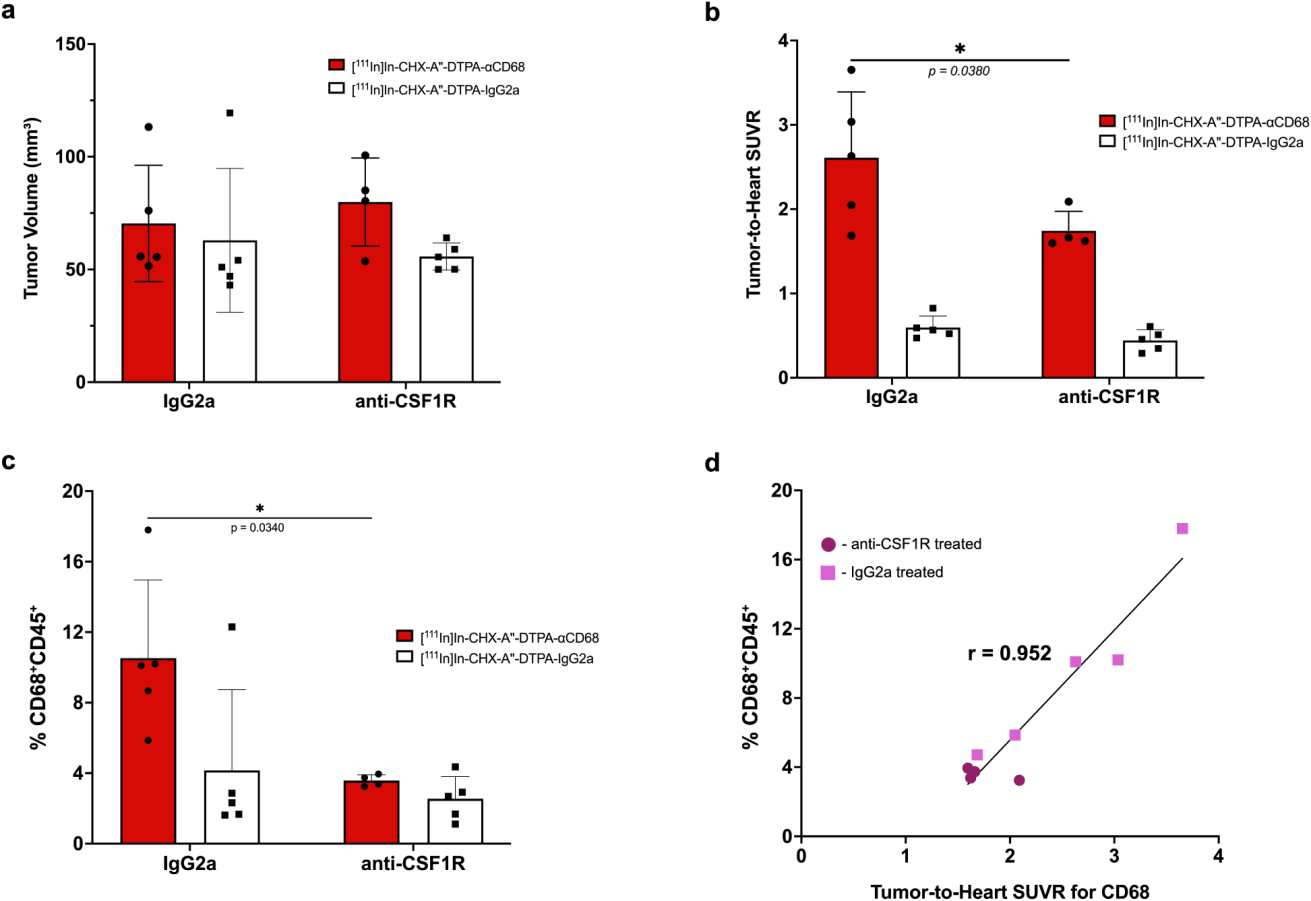
ImmunoSPECT after anti-CSF1R or isotype control treatment: (a) Final tumor volumes after treatment with anti-CSF1R or IgG2a control (b) Comparison of tumor-to-heart SUVR for [^111^In]­In-CHX-A″-DTPA-αCD68 and [^111^In]­In-CHX-A″-DTPA-IgG2a between anti-CSF1R and IgG2a treatment groups (*n* = 4 – 5 per group). (c) Expression of cell surface mCD68 by flow cytometry, quantified as % CD68^+^CD45^+^ live cells. (d) Pearson correlation of SUVR and % CD68^+^CD45^+^ for anti-CSF1R and IgG2a-treated mice imaged with [^111^In]­In-CHX-A″-DTPA-αCD68 (*r* = 0.952).

## Discussion

We developed [^111^In]­In-CHX-A″-DTPA-αCD68 to noninvasively quantify total TAM burden in vivo, targeting CD68—an established clinical biomarker for pan-macrophages. Despite about 72% sequence conservation between human and mouse CD68,^11^ the development of a noninvasive imaging probe for mCD68 had not previously been reported until the current study. A plausible reason is the predominantly intracellular localization of CD68; however, CD68 rapidly shuttles to the cell surface, making it accessible to antibody-based targeting. Here, we demonstrate that [^111^In]­In-CHX-A″-DTPA-αCD68 binds specifically to CD68^+^ macrophages in vitro (Figure 2) and in vivo (Figures 3 and 4), confirming that cell surface CD68 is a viable target for immunoSPECT imaging. Although a statistically significant difference in radioligand binding was observed between nonblocking and CD68-blocking conditions on Renca cells in vitro (Figure 2c), absolute binding was approximately 6-fold lower than on BMDMs, indicating minimal CD68 expression on Renca cells. Surface expression of CD68 on live BMDMs in vitro (Figure 1c–e) and on TAMs ex vivo (Figures 4 and 6) was confirmed by flow cytometry, consistent with prior reports for both mCD68 and human CD68.^10,11^

Several nuclear imaging probes have been developed targeting different macrophage biomarkers. TSPO has long been used as an indirect measure of macrophage activity in neuroinflammation; however, TSPO is expressed on multiple cell types other than macrophages, and genetic polymorphisms in different individuals impact the binding affinity of these probes.^22^ Positron emission tomography (PET) and SPECT imaging probes have been developed to target CD206 or CD163 as biomarkers for immunosuppressive subtypes of macrophages.^23,24^ Another probe targeted F4/80 with [^111^In]­In-DTPA-αF4/80-A3–1 for the pro-inflammatory subtype of macrophages in mice.^25^ Notably, imaging with this tracer was validated by histopathological analysis of CD68 expression in tissues. However, F4/80 is not expressed on human macrophages, and thus cannot be translated to humans. PET tracers for CD11b and MHC II were developed, but these biomarkers lack specificity for macrophages given the expression of these biomarkers in other immune cells, including monocytes, B cells, and/or dendritic cells.^26,27^ Finally, ^64^Cu-CD68-Fc is a mouse fusion protein in which the CD68 ectodomain serves as the targeting moiety for oxidized LDL—an indirect approach that lacks specificity for macrophages, as scavenger receptors are also expressed on dendritic and endothelial cells.^28^ Thus, these targets discussed above are limited to one or more of the following: only present in mouse macrophages and absent in human macrophages, expressed in other immune cells, or only capture immunosuppressive macrophages. Given the plasticity of macrophage function, we posit that imaging pan-macrophages would contribute to the limited tools available for molecular imaging of these immune cells.^29^ Pairing [^111^In]­In-CHX-A″-DTPA-αCD68 with other imaging probes described above would strengthen the specificity of imaging readouts for the different functions of macrophages.

We evaluated the specificity of [^111^In]­In-CHX-A″-DTPA-αCD68 for CD68 in a syngeneic tumor model of RCC, Renca. The syngeneic setting is a necessity for immune cell imaging probe development. Only in an immunocompetent host can the full innate and adaptive immune response be preserved, enabling accurate assessment of macrophage dynamics, immune-relevant treatment effects, and potential immunological consequences of probe administration. Xenograft models in immunodeficient mice are fundamentally unsuited for this purpose. While our syngeneic studies employed an anti-mCD68 probe, the underlying macrophage biology—including CD68 expression dynamics, macrophage recruitment to tumors, and CSF1R-dependent macrophage depletion—is conserved between mouse and human.^11^ The syngeneic animal model therefore provides a complete immune system in which to establish proof-of-concept for CD68-targeted imaging and macrophage-modulating treatments, with direct mechanistic relevance to the human disease setting.

High expression of CD68^+^ TAMs in RCC patients is correlated with significantly lower survival.^30^ Renca is a widely used syngeneic model for RCC studies, since it mimics disease progression and immune microenvironment composition in humans; it is extensively used in the preclinical development of investigational immunotherapies, especially those that focus on innate immunity.^31–34^ In this model, we compared the uptake of [^111^In]­In-CHX-A″-DTPA-αCD68 with that of its isotype control, [^111^In]­In-CHX-A″-DTPA-IgG2a (Figure 3). Comparison with an isotype control is an established approach for confirming target specificity in immune cell imaging.^25,35,36^ Since SPECT imaging lacks the sensitivity of PET, quantification of SPECT images using absolute SUV values may not be reliable.^37^ Therefore, we quantified uptake by normalizing segmentations to a reference organ (heart) and reported this ratio as SUVR. The strong correlation between tumor-to-heart SUVR and ex vivo CD68^+^CD45^+^ cells by flow cytometry (*r* = 0.854) confirms that immunoSPECT signal in the TME reflects CD68^+^ macrophage content rather than tumor cell-associated uptake in vivo. Ex vivo flow cytometry analysis of tumor and spleen cells confirmed cell surface CD68 expression and, importantly, showed no significant differences in CD68 expression between probes when normalized to viable cells (Figure 4b), indicating that the injected dose of [^111^In]­In-CHX-A″-DTPA-αCD68 does not deplete CD68^+^ macrophages in vivo. This finding was further supported by comparable CD68^+^ TAM levels in tumor-bearing mice that did not receive ^111^In-labeled antibody (*p* > 0.05; Supplementary Figure S4).

Biodistribution studies showed significantly higher uptake of [^111^In]­In-CHX-A″-DTPA-αCD68 in the tumor, spleen, and bone marrow compared with the radiolabeled isotype control (Figure 5). Elevated uptake in the spleen and bone marrow is expected, as these organs are primary sites of macrophage differentiation.^24,25^ This result is consistent with high cell surface CD68 expression confirmed by flow cytometry for the tumor and spleen (Figure 4b). Low uptake in other normal organs, such as the lungs, liver, kidneys, and heart, is consistent with the Human Protein Atlas.^38^ The absence of brain uptake reflects the inability of conventional antibodies to cross an intact blood–brain barrier.^39^ Despite the presence of macrophages in normal organs, our mCD68 probe showed preferential uptake in the TME. These results confirm that CD68^+^ cells are present at higher density in solid tumors than in most normal organs, with the exception of secondary lymphoid organs (e.g., spleen), supporting CD68 as a viable imaging biomarker for TAMs. While CD68 is expressed intracellularly across a broader range of myeloid cells,^11^ cell-surface CD68 expression is substantially enriched upon monocyte-to-macrophage differentiation (Figure 1c–e), with the lowest expression at day 1 and the highest at day 3 postdifferentiation. This surface-restricted expression pattern supports the selectivity of our probe for mature macrophages rather than the broader myeloid compartment. We acknowledge that the contribution of nonmacrophage myeloid cells to the imaging signal cannot be fully excluded. However, the strong correlation between the in vivo tracer uptake and ex vivo CD68 quantification, together with favorable tumor-to-background ratios, supports that the dominant signal reflects CD68-expressing macrophages.

Having established probe specificity, we evaluated whether [^111^In]­In-CHX-A″-DTPA-αCD68 is sensitive to treatment-induced changes in TAM burden in vivo. In Renca tumor-bearing mice treated with the macrophage-depleting agent anti-CSF1R, tumor-to-heart SUVR was 40% lower than in IgG controls (*p* = 0.0380; Figure 6b), despite no difference in tumor volumes (Figure 6a). Concordantly, ex vivo flow cytometry showed significantly lower % CD68^+^ CD45^+^ TAMs in anti-CSF1R-treated mice (Figure 6c), and tumor-to-heart SUVR correlated very strongly with surface CD68 expression (*r* = 0.952; Figure 6d). Together, these results demonstrate that [^111^In]­In-CHX-A″-DTPA-αCD68 can noninvasively quantify TAM depletion in vivo, independent of changes in tumor volume.

A limitation of this study is that animal numbers in some cohorts were reduced due to poor tumor engraftment or death prior to imaging. Additionally, the current probe targets mCD68 and is not cross-reactive with human CD68; the development of an anti-human CD68 imaging probe to enable clinical translation is an area of active investigation by our group. Future work involves the development of a radiotracer for use in humans and an in-depth analysis of the immune phenotypes and the function of macrophages. We can also apply this probe to other inflammatory conditions beyond cancer, where macrophages can impact disease progression, such as rheumatoid arthritis, Alzheimer’s disease, and cardiovascular disease.^40^

## Conclusions

We report the first immunoSPECT imaging agent targeting mCD68, validated here as a noninvasive biomarker of total TAM burden in a syngeneic RCC model. [^111^In]­In-CHX-A″-DTPA-αCD68 demonstrated specific binding to CD68^+^ macrophages in vitro and in vivo, with immunoSPECT signal strongly correlated with ex vivo CD68 expression and sensitive to treatment-induced TAM depletion. Whole-body three-dimensional imaging revealed heterogeneous intratumoral macrophage distribution, underscoring the advantage of noninvasive volumetric quantification over biopsy-based approaches. This probe is well-suited for mechanistic preclinical studies requiring longitudinal TAM tracking and for evaluating the translational potential of CD68-targeted SPECT imaging. This imaging probe could be applied to inflammatory diseases beyond oncology, to understand macrophage infiltration as a mechanism of disease progression.

## Supplementary Material



## Data Availability

All data generated or analyzed during this study are included in this published article and its Supporting Information files. Imaging data will be made publicly available upon reasonable request from the corresponding author.

## Notes

Workflow for isolation of bone marrow derived macrophages (BMDM); representative flow cytometry gating strategy; confirmation of radiochemical yield and purity for [^111^In]­In-CHX-A″-DTPA-αCD68; mCD68 MFI in CD45^+^ single cells of tumor tissues; biodistribution study showing uptake by sex; and tumor growth curve for Renca allograft model treated with anti-CSF1R and IgG2a (PDF)

## Abbreviations

TAM: Tumor-Associated Macrophages
PET: Positron Emission Tomography
SPECT: Single Photon Emission Computed Tomography
mCD68: Mouse CD68
BMDMs: Bone Marrow-Derived Macrophages
rMCSF: Recombinant Macrophage Colony-Stimulating Factor
PBS: Phosphate-Buffered Saline
PFA: Paraformaldehyde
BSA: Bovine Serum Albumin
PBST: PBS Containing 0.05% Tween-20
DAPI: 4′,6-diamidino-2-phenylindole
CHX-A″-DTPA: [(R)-2-Amino-3-(4-isothiocyanatophenyl)­propyl]-trans-(S,S)-cyclohexane-1,2-diamine-pentaacetic acid
Radio-iTLC: Radio-Instant Thin-Layer Chromatography
Radio-SEC-HPLC: Radio-Size-Exclusion High-Performance Liquid Chromatography
K_D_: Binding Affinity
IACUC: Institutional Animal Care and Use Committee
% ID/g: Percentage of Injected Dose per Gram of Organ
THR: Tumor-to-Heart Ratio
SUVR: Standardized Uptake Value Ratio
HI_SUV_: SUV heterogeneity index
MFI: Median Fluorescence Intensity

## References

[1] Yang S., Zhao M., Jia S. (2023). Macrophage: Key player in the pathogenesis
of autoimmune diseases. Front. Immunol.

[2] Liu H., Lv Z., Zhang G., Yan Z., Bai S., Dong D., Wang K. (2024). Molecular understanding
and clinical aspects of tumor-associated
macrophages in the immunotherapy of renal cell carcinoma. J. Exp. Clin. Cancer Res..

[3] Santoni M. (2013). Emerging role of tumor-associated macrophages as therapeutic
targets
in patients with metastatic renal cell carcinoma. Cancer Immunol., Immunother.

[4] Rannikko J. H., Hollmén M. (2024). Clinical landscape
of macrophage-reprogramming cancer
immunotherapies. Br. J. Cancer.

[5] Perry C. J. (2018). Myeloid-targeted immunotherapies act in synergy to induce inflammation
and antitumor immunity. J. Exp. Med..

[6] Weiss S. A. (2021). A Phase I Study of APX005M and Cabiralizumab with or without Nivolumab
in Patients with Melanoma, Kidney Cancer, or Non–Small Cell
Lung Cancer Resistant to Anti-PD-1/PD-L1. Clin.
Cancer Res..

[7] Djureinovic D., Weiss S.A., Krykbaeva I., Qu R., Vathiotis I., Moutafi M., Zhang L., Perdigoto A.L., Wei W., Anderson G. (2023). A bedside to bench study of anti-PD-1,
anti-CD40, and anti-CSF1R indicates that more is not necessarily better. Mol. Cancer.

[8] Ostuni R. (2015). Macrophages and cancer:
from mechanisms to therapeutic implications. Trends Immunol..

[9] Gottfried E. (2008). Expression of CD68 in Non-Myeloid Cell Types. Scand. J. Immunol.

[10] Ramprasad M. P., Terpstra V., Kondratenko N., Quehenberger O., Steinberg D. (1996). Cell surface expression
of mouse macrosialin
and human CD68 and their role as macrophage receptors for oxidized
low density lipoprotein. Proc. Natl.
Acad. Sci. U. S. A..

[11] Chistiakov D. A. (2017). CD68/macrosialin: not just a histochemical
marker. Lab. Invest.

[12] Caobelli F. (2016). Simultaneous dual-isotope
solid-state detector SPECT for improved
tracking of white blood cells in suspected endocarditis. Eur. Heart J..

[13] Mendoza R. (2022). Mouse Bone Marrow Cell
Isolation and Macrophage Differentiation. Methods
Mol. Biol..

[14] Luo L., Hu X., Dixon M.L., Pope B.J., Leavenworth J.D., Raman C., Meador W.R., Leavenworth J.W. (2021). Dysregulated
follicular regulatory T cells and antibody responses exacerbate experimental
autoimmune encephalomyelitis. J. Of Neuroinflammation.

[15] Wang J. (2017). UV-induced somatic mutations
elicit a functional T cell response
in the YUMMER1.7 mouse melanoma model. Pigm.
Cell Melanoma Res..

[16] Cavaliere A. (2021). Development of [89Zr]­ZrDFO-amivantamab bispecific to EGFR and c-MET
for PET imaging of triple-negative breast cancer. Eur. J. Nucl. Med. Mol. Imaging.

[17] Andreeva E. R., Pugach I. M., Orekhov A. N. (1997). Subendothelial
smooth muscle cells
of human aorta express macrophage antigen in situ and in vitro. Atherosclerosis.

[18] Syed A. K. (2019). Characterizing Trastuzumab-Induced
Alterations in Intratumoral Heterogeneity
with Quantitative Imaging and Immunohistochemistry in HER2+ Breast
Cancer. Neoplasia.

[19] Miyashi Y. (2025). Heterogeneity of Tumor
Glucose Metabolism in Schwannomas Between
Trunk and Extremities: An Imaging Study. Vivo.

[20] Tran T. T., Caulfield J., Zhang L., Schoenfeld D., Djureinovic D., Chiang V.L., Oria V., Weiss S.A., Olino K., Jilaveanu L.B. (2023). Lenvatinib or anti-VEGF in combination
with anti–PD-1 differentially augments antitumor activity in
melanoma. JCI Insight.

[21] O’Brien S. A. (2021). Activity of tumor-associated
macrophage depletion by CSF1R blockade
is highly dependent on the tumor model and timing of treatment. Cancer Immunol., Immunother.

[22] Wu C. (2014). Longitudinal PET Imaging
of Muscular Inflammation Using 18F-DPA-714
and 18F-Alfatide II and Differentiation with Tumors. Theranostics.

[23] Blykers A. (2015). PET Imaging of Macrophage Mannose Receptor–Expressing Macrophages
in Tumor Stroma Using 18F-Radiolabeled Camelid Single-Domain Antibody
Fragments. J. Nucl. Med..

[24] Lauwers Y., De Groof T. W. M., Vincke C., Van Craenenbroeck J., Jumapili N. A., Barthelmess R. M., Courtoy G., Waelput W., De Pauw T., Raes G. (2024). Imaging of tumor-associated
macrophage dynamics during immunotherapy using a CD163-specific nanobody-based
immunotracer. Proc. Natl. Acad. Sci. U. S. A..

[25] Terry S. Y. A. (2015). 111In-anti-F4/80-A3–1
antibody: a novel tracer
to image macrophages. Eur. J. Nucl. Med. Mol.
Imaging.

[26] Nigam S. (2020). Preclinical ImmunoPET Imaging of Glioblastoma-Infiltrating
Myeloid
Cells Using Zirconium-89 Labeled Anti-CD11b Antibody. Mol. Imaging Biol..

[27] Rashidian M. (2015). Noninvasive imaging
of immune responses. Proc.
Natl. Acad. Sci. U. S. A..

[28] Bigalke B. (2014). PET/CT and MR imaging
biomarker of lipid-rich plaques using [64Cu]-labeled
scavenger receptor (CD68-Fc). Int. J. Cardiol..

[29] Jayasingam S. D., Citartan M., Thang T.H., Mat Zin A.A., Ang K.C., Ch’ng E.S. (2020). Evaluating the Polarization of Tumor-Associated Macrophages
Into M1 and M2 Phenotypes in Human Cancer Tissue: Technicalities and
Challenges in Routine Clinical Practice. Front.
Oncol.

[30] Dannenmann S. R. (2013). Tumor-associated macrophages
subvert T-cell function and correlate
with reduced survival in clear cell renal cell carcinoma. OncoImmunology.

[31] Sobczuk P. (2020). Choosing The Right Animal
Model for Renal Cancer Research. Transl. Oncol.

[32] Yu J. W. (2018). Tumor-immune profiling
of murine syngeneic tumor models as a framework
to guide mechanistic studies and predict therapy response in distinct
tumor microenvironments. PLoS One.

[33] Rayman P., Diaz-Montero C. M., Yang Y., Rini B., Elson P., Finke J. (2015). Modulation of immune cell infiltrate with sunitinib
to improve anti-PD1 therapy in preclinical tumor model. J. Immunotherapy Cancer.

[34] Zhang X. (2019). Combination immunotherapy
with interleukin-2 surface-modified tumor
cell vaccine and programmed death receptor-1 blockade against renal
cell carcinoma. Cancer Sci..

[35] Put S. (2013). SPECT Imaging of Joint
Inflammation with Nanobodies Targeting the
Macrophage Mannose Receptor in a Mouse Model for Rheumatoid Arthritis. J. Nucl. Med..

[36] Falk I. N., Chaney A.M., Verma R., Kuo R.C., Reyes S., Carlson M., Kalita M., Azevedo C., Jackson I.M., Green J., Alam I.S. (2026). TREM1-PET imaging maps
whole-body innate immune responses in a mouse model of metastatic
melanoma. Sci. Rep..

[37] Dickson J., Ross J., Vöö S. (2019). Quantitative
SPECT: the time is now. EJNMMI Phys..

[38] Berglund L. (2008). A Genecentric Human Protein Atlas for Expression
Profiles Based on
Antibodies. Mol. Cell. Proteomics.

[39] Pardridge W. M. (2023). Receptor-mediated
drug delivery of bispecific therapeutic antibodies through the blood-brain
barrier. Front. Drug Deliv..

[40] Chi Y., Jiang H., Yin Y., Zhou X., Shao Y., Li Y., Rao J. (2025). Macrophage Signaling Pathways in Health and
Disease: From Bench to Bedside Applications. MedComm.

