# Supplementary material for: Development of [111In]In-CHX-A″-DTPA-αCD68 for ImmunoSPECT to Image Murine Macrophages
Source: Mol Pharm. 2026 Jul 2;23(8):4079–88. doi: 10.1021/acs.molpharmaceut.5c01902 (PMC13439659; doi:10.1021/acs.molpharmaceut.5c01902)
Supplement: Supplementary file 1 [file mp5c01902_si_001.pdf]

Supplementary Information

Title: Development of [^{111}In]In-CHX-A”-DTPA-αCD68 for immunoSPECT to image murine macrophages

Authors & Affiliations: Anna Strong,[1*] Erika Belitzky,[2*] Ayla Vaughn Embs,[1*] Samantha Katz,[2] Alessandra Cavaliere,[2] Dijana Djureinovic,[3] Borna Roohani,[1] Michael Liu,[2] Carla Rothlin,[4] Jianmei W. Leavenworth,[5,6] Lucia Jilaveanu,[3] Harriet M. Kluger,[3] and Bernadette Marquez-Nostra[1,6#]

[*] Authors contributed equally to this work

[1]Department of Radiology, University of Alabama at Birmingham, Birmingham, AL USA 35233

[2]Yale PET Center, Department of Radiology and Biomedical Imaging, Yale University, New Haven, CT, USA 06520

[3]Department of Medical Oncology, Yale University, New Haven, CT, USA 06520

[4]Department of Laboratory Medicine and Pathology, University of Minnesota, Minneapolis, MN, USA 55455

[5]Department of Neurosurgery, University of Alabama at Birmingham, Birmingham, AL USA 35233

[6]The O’Neal Comprehensive Cancer Center, University of Alabama at Birmingham, Birmingham, AL USA 35233

[#] Correspondence: bmnostra@uab.edu, Phone: (205) 934-2950

Running Title: ImmunoSPECT imaging of murine macrophages

Manuscript Category: Original Research

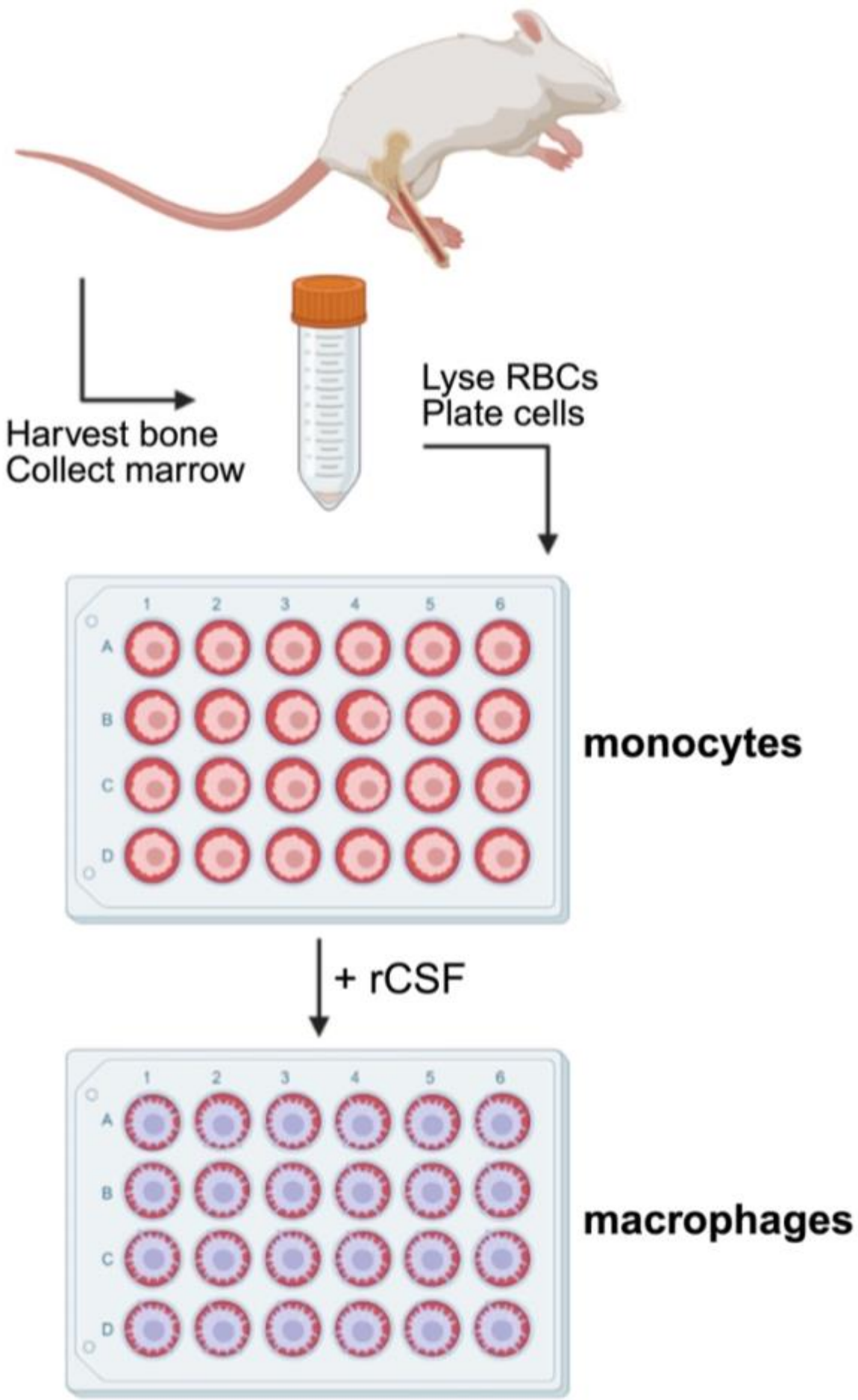

Figure S1. (a) Workflow for isolation of bone marrow derived macrophages (BMDM). Created in BioRender. Strong, A. E. (2026) https://BioRender.com/2648w99

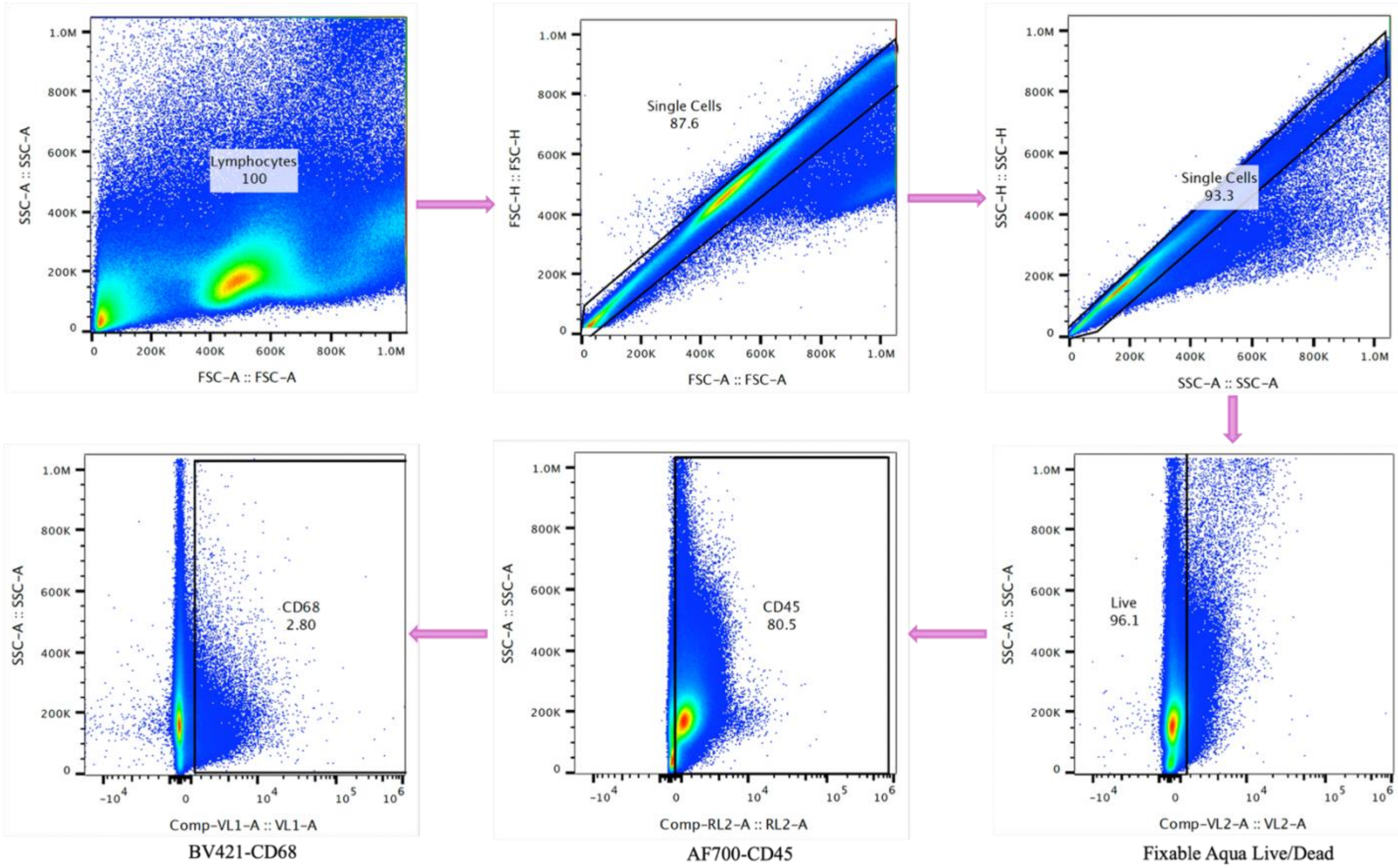

Figure S2. Representative flow cytometry gating strategy starting from total cells then isolating single cells, live cells, CD45$^+$ immune cells and then finally cell surface CD68$^+$ cells.

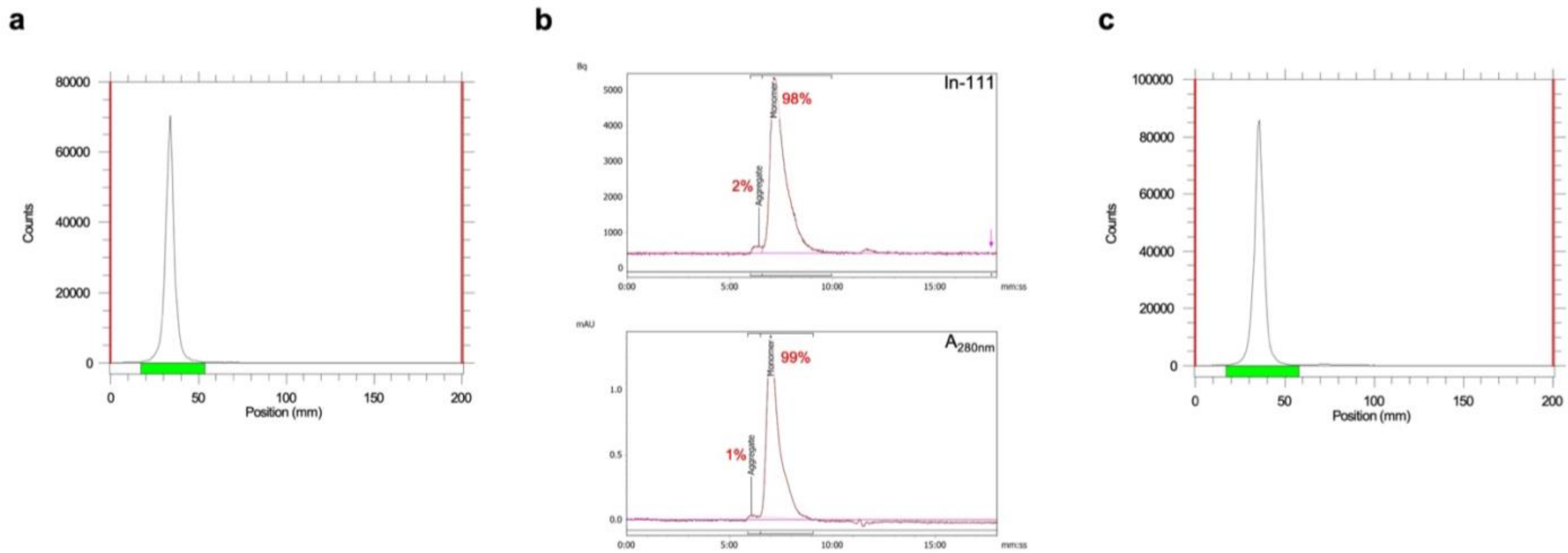

Figure S3. Confirmation of (a) radiochemical yield for [^{111}In]In-CHX-A''-DTPA-αCD68 via radio-TLC, (b) radiochemical purity for [^{111}In]In-CHX-A''-DTPA-αCD68 via radio-SEC-HPLC. Shown are the chromatograms for In-111 (top) and A280nm (bottom), and (c) radiochemical yield for [^{111}In]In-CHX-A''-DTPA-IgG via radio-TLC.

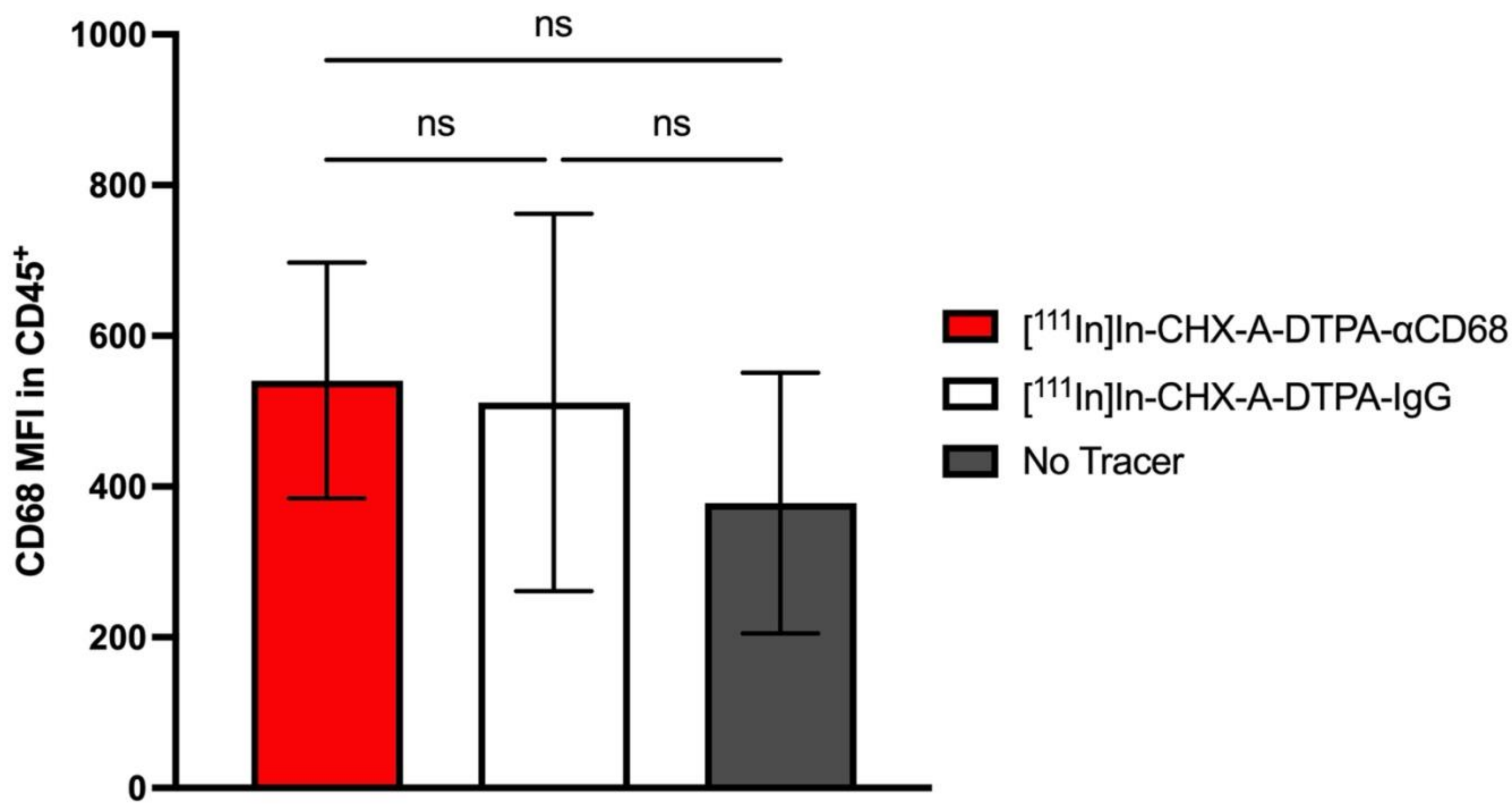

Figure S4. CD68 MFI in CD45+ tumor single cells demonstrating no differences between tumors from mice injected with ^{111}In-labeled antibodies or no tracer injection.

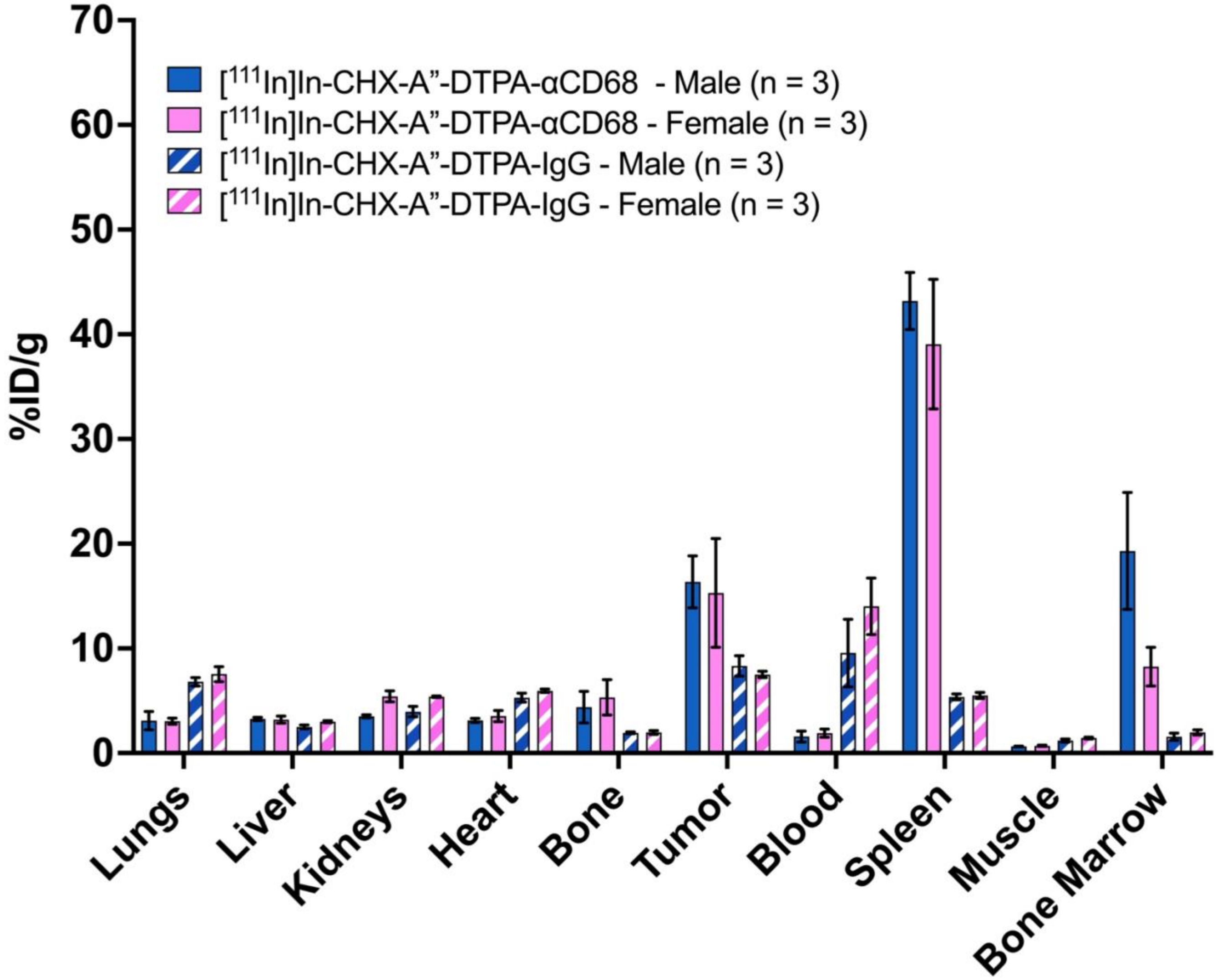

Figure S5. Biodistribution study of [^{111}In]In-CHX-A"-DTPA-αCD68 and [^{111}In]In-CHX-A"-DTPA-IgG2a in Renca allograft models showing uptake between sexes.

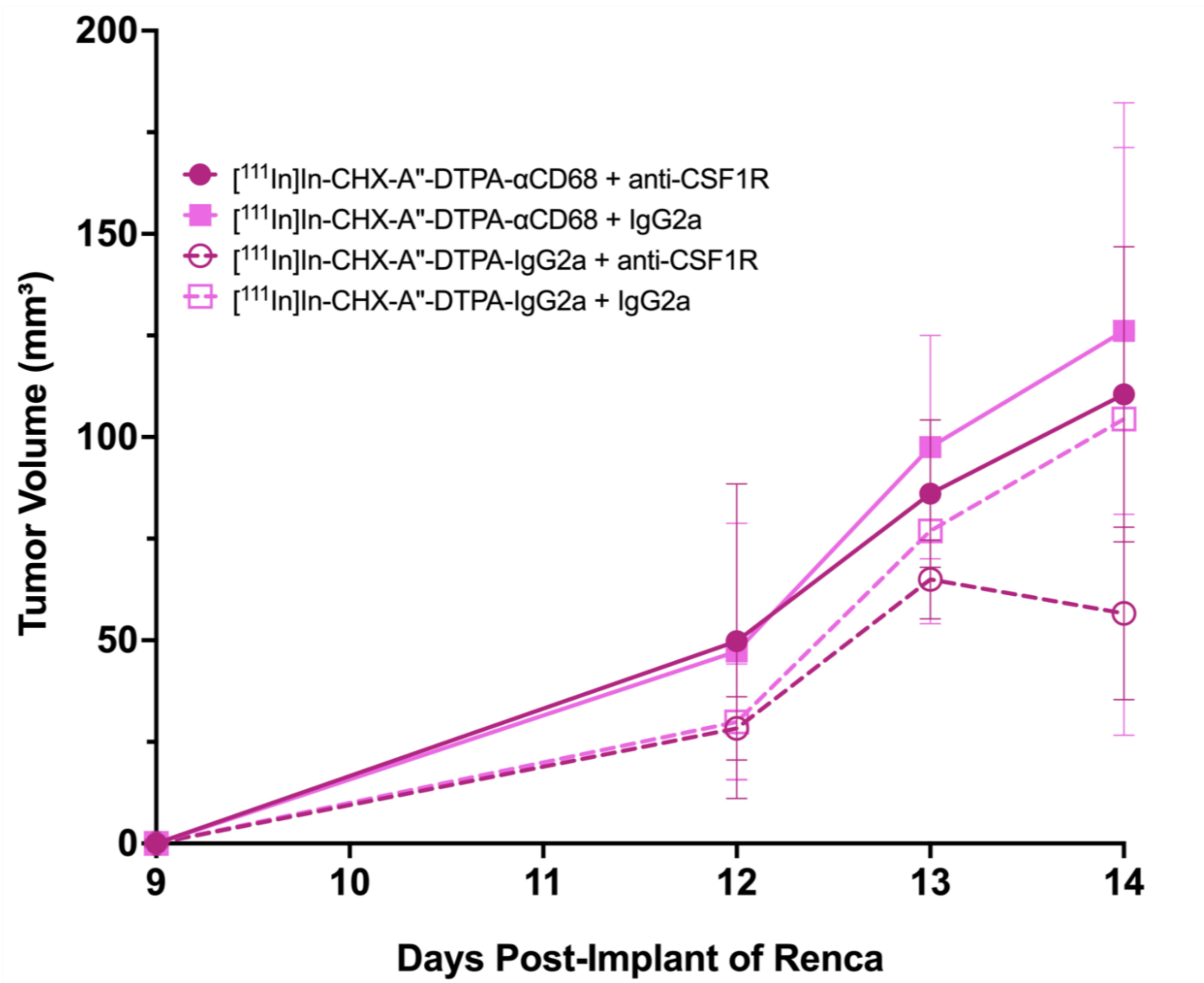

Figure S6. Tumor growth curve for Renca allograft model treated with anti-CSF1R and IgG2a beginning the day of inoculating Renca cells. ImmunoSPECT imaging was performed at 14 days thereafter.
